# Some Classes of Logarithmic-Type Imputation Techniques for Handling Missing Data

**DOI:** 10.1155/2021/8593261

**Published:** 2021-12-20

**Authors:** Awadhesh K. Pandey, G. N. Singh, D. Bhattacharyya, Abdulrazzaq Q. Ali, Samah Al-Thubaiti, H. A. Yakout

**Affiliations:** ^1^Department of Mathematics, School of Physical Sciences, DIT University, Dehradun, Uttarakhand 248 009, India; ^2^Department of Mathematics & Computing, Indian Institute of Technology (ISM), Dhanbad 826 004, Jharkhand, India; ^3^Mharat Academy for Training & Development, Ibb, Yemen; ^4^Department of Mathematics and Statistics, College of Science, Taif University, P.O. Box 11 099, Taif 21 944, Saudi Arabia; ^5^Department of Physics, College of Science, King Khalid University, PO Box 9004, Abha 61 413, Saudi Arabia

## Abstract

In this manuscript, three new classes of log-type imputation techniques have been proposed to handle missing data when conducting surveys. The corresponding classes of point estimators have been derived for estimating the population mean. Their properties (Mean Square Errors and bias) have been studied. An extensive simulation study using data generated from normal, Poisson, and Gamma distributions, as well as real dataset, has been conducted to evaluate how the proposed estimator performs in comparison to several contemporary estimators. The results have been summarized, and discussion regarding real-life applications of the estimator follows.

## 1. Introduction

Any project has several constraints involved, such as budget restrictions, time limitations, and deadlines. As a result, it is not feasible to study the entire population, and sampling is indispensable for any field of study [1–4]. Sampling has immense applications in various industries such as manufacturing and quality control. It can be utilized to gather information on the notable characteristics of items, such as electrical appliances and household appliances, machine parts like screws and bolts, automobiles, and computer parts like chips. Sampling also has applications in environmental problems that require the estimation of physical, geographical, economical, and other characteristics, before data analysis can begin [5, 6]. Mean, median, variance, and other statistics are essential for studies involving various environmental parameters, such as estimation of the amount of rainfall received in an area prone to droughts and the air quality of a city with high traffic density. Sample surveys may be designed to collect such information.

Missing data is a frequent element in sample surveys and is a primary contributor towards decline of data quality and incorrect inferences. Hence, it is crucial that survey statisticians deal with the stochastic nature of such incomplete data. It is essential to understand the assumptions which have to be made and the methods that can be utilized to deal with the problem of ignorability of completeness mechanism. The authors of [7, 8] and many others have studied the mechanisms of missing data. Of these, the ones that are most relevant to the survey literature are missing completely at random (MCAR), missing at random (MAR), and missing not at random (MNAR). When data is missing randomly or by chance, MCAR is said to occur. MAR occurs when the missingness does not depend on the variable under study (which may be unobserved), but on some other variable (which is fully observed). MNAR occurs when missingness depends on the variable under study.

A number of statistical techniques have been developed over the past decades to handle the situation of missing data. The study in [9] was the first to suggest that a subsample of nonrespondents be contacted again by mail surveys. Another widely employed technique is imputation, in which a suitable function of the variables is used to fill in the missing values. This ensures the completeness of the sample in terms of structure prior to the commencement of statistical analysis. Some popular imputation methods include mean, regression, hot deck, cold deck, and nearest neighbor methods of imputation, among others. Imputation techniques in the survey literature are due to [10–27], among others.

Information from an auxiliary variable can be utilized to provide an improved estimate for population characteristics. Such information may be readily available as secondary data from previous surveys or census or may be collected during the survey procedure at little to no additional cost. Some examples of such auxiliary information include the lifetime of a previous batch of bulbs when studying the life of a current lot of bulbs and the speed of cars when studying the mileage of cars.

This manuscript proposed three novel logarithmic-type imputation methods to neutralize the nuisance effects of nonresponse in survey sampling. The corresponding classes of point estimators that may be used for estimating population mean have been studied in detail. The subsequent sections of the manuscript are devoted to the theoretical analysis of the properties of the proposed estimators, in terms of bias and Mean Square Error (MSE), and empirical study to examine the performance of the proposed estimators in comparison with some contemporary estimators, based on both simulated data and real data, and the conclusions have been presented. These are structured as follows: Sections 2 and 3 introduce the sample structure and notations and some conventional estimators of population mean, respectively, which have been used subsequently in the manuscript.  Section 4 introduces the proposed classes of estimators, and comments on its existence, consistency, properties, and implementation in R. The empirical study involving simulated data and real data have been presented in Sections 5 and 6, respectively.  Section 7 summarizes the main findings and conclusions.

## 2. Sampling Scheme and Notations Used

Let the characteristic of interest be denoted by *Y*. A correlated auxiliary variable *X* with the availability of complete information on it and known population mean is considered.

The sample structure as well as the notations used in the subsequent sections of the manuscript have been introduced in Table 1.

## 3. Some Conventional Estimators

It is crucial to conduct thorough literature review and examine the properties of some existing estimators of population mean, before new estimators can be proposed. A few such estimators have been discussed in this section.

The mean estimator is a simple and widely used estimator, which provides an estimate of the population mean using the average of the responses. Ratio estimator improves over the mean estimator by utilizing auxiliary information on a correlated variable. Numerous other estimators which make effective use of auxiliary information have been developed, for instance, the estimator proposed in [28] and regression-type estimators proposed in [29], among others.

The structures of some of these estimators have been given in Table 2, while the expressions for their respective variances (V) or Mean Square Errors (MSEs) have been given in Table 3.

It is to be noted that most conventional estimators make use of simple functional forms, such as linear combinations, exponential functions, and chains. Logarithmic functions are rarely seen. This can be partially attributed to computational limitations associated with such functions. However, the advent of supercomputers and improvement in computational powers have eliminated such obstacles. Logarithms are useful because they express numbers in a reasonable scale that is easy to understand by people. Logarithms count multiplication as steps and hence can express events whose magnitudes can vary in a drastic manner, such as earthquakes, on a singular scale that has a compact range. Logarithmic-scale graphs are efficient in graphically depicting such widely varying magnitudes in a single scale. In log-scale graphs, straight lines often represent exponential changes, thus making them easier to interpret. Some real-life examples of use of logarithms are decibels for measuring sound, Richter scale for measuring earthquakes, pH scale for measuring acidity, etc. Logarithms can also be used to study exponential growth and decay, such as bacterial growth in a Petri dish, interest rates (the implicit growth rate), and radioactive decay in radiocarbon dating. Hence, it is reasonable to explore the use of log-type estimators for estimation of various population parameters. This has been the motivation behind the construction of the proposed classes of logarithmic-type estimators.

## 4. Formulation of the Proposed Classes of Logarithmic-Type Estimators

Let *B*_*i*_ where *B*=*Y* or *B*=*X* denote, respectively, the values for the *i*^*th*^ population unit of characteristics *Y* and *X*. Let *A* and *A*^*c*^ denote the sets of respondents and nonrespondents, respectively. The following imputation methods may be suggested to deal with the problem of missing data:

(1) $$\begin{array}{c} y_{1i}=\left\{\begin{array}{cc} y_{i}, & \text{if }i\in A,\\ {\bar{y}}_{r}+\frac{\alpha nx_{i}}{\left(n-r\right){\bar{x}}_{n-r}}\log\left(\frac{{\bar{x}}_{n}}{\bar{X}}\right), & \text{if }i\in A^{c}, \end{array}\right. \end{array}$$

(2) $$\begin{array}{c} y_{1i}=\left\{\begin{array}{cc} y_{i}, & \text{if }i\in A,\\ {\bar{y}}_{r}+\frac{\beta nx_{i}}{\left(n-r\right){\bar{x}}_{n-r}}\log\left(\frac{{\bar{x}}_{r}}{\bar{X}}\right), & \text{if }i\in A^{c}, \end{array}\right. \end{array}$$

(3) $$\begin{array}{c} y_{1i}=\left\{\begin{array}{cc} y_{i}, & \text{if }i\in A,\\ {\bar{y}}_{r}+\frac{\gamma nx_{i}}{\left(n-r\right){\bar{x}}_{n-r}}\log\left(\frac{{\bar{x}}_{n}}{{\bar{x}}_{r}}\right), & \text{if }i\in A^{c}, \end{array}\right. \end{array}$$

where *α*, *β*,  and *γ* are constants, to be determined in such a way that they minimize the MSE.

The point estimator under an imputation method is given in

(4) $$\begin{array}{c} T=\frac{1}{n}\sum_{i\in S}y_{\cdot i}=\frac{1}{n}\left[\sum_{i\in R}y_{\cdot i}+\sum_{i\in R^{c}}y_{\cdot i}\right]. \end{array}$$

Using Equation (4), under the imputation outlined in Equations (1)–(3), respectively, the expressions for the corresponding classes of logarithmic-type point estimators of $\bar{Y}$ are obtained as

(5) $$\begin{array}{c} T_{1}={\bar{y}}_{r}+\alpha \;\log\left(\frac{{\bar{x}}_{n}}{\bar{X}}\right), \end{array}$$

(6) $$\begin{array}{c} T_{2}={\bar{y}}_{r}+\beta \;\log\left(\frac{{\bar{x}}_{r}}{\bar{X}}\right), \end{array}$$

(7) $$\begin{array}{c} T_{3}={\bar{y}}_{r}+\gamma \;\log\left(\frac{{\bar{x}}_{n}}{{\bar{x}}_{r}}\right). \end{array}$$

### 4.1. Existence and Consistency of the Estimator

The domain of values for which an estimator exists should be specified, so that survey statisticians or those working in the field are able to determine whether it is reasonable to use an estimator in a practical scenario.

The proposed classes of estimators consist of the log(*x*) function, which exists for all positive values of *x*. Hence, *T*_*i*_, *i*=1,2,3, exist for all positive values of *x*.

Hence, the proposed estimators can be used for all real, positive values of the characters under study. For real-world scenarios, many characters of interest take only positive values. For example, measurements such as length, breadth, height, weight, diameter, currencies, and number of an item do not take negative values. Hence, the proposed estimator can be used in such practical scenarios.

It is to be noted that the structure of the estimator is consistent for large-sample approximations. As *n*⟶*∞*, ${\bar{y}}_{r}⟶\bar{Y}$, ${\bar{x}}_{r}⟶\bar{X}$, and ${\bar{x}}_{n}⟶\bar{X}$, log(1)=0. Hence, $T_{i}⟶\bar{Y}$, for *i*=1,2,3.

### 4.2. Properties of the Proposed Estimator

Various properties can be used to measure the “goodness” of an estimator. Two such properties, namely, bias and Mean Squared Error (MSE), have been discussed in this manuscript. Bias paints a picture of the expected deviation from the true value of a parameter, while MSE gives an idea about the degree of spread. Large-sample assumptions have been considered for the purpose. The expressions have been derived up to the first order of approximations. Some transformations involving error terms have been employed for the purpose, given as follows:

(8) $$\begin{array}{c} \eta_{0}=\frac{{\bar{y}}_{r}-\bar{Y}}{\bar{Y}},\\ \eta_{1}=\frac{{\bar{x}}_{r}-\bar{X}}{\bar{X}},\\ \eta_{2}=\frac{{\bar{x}}_{n}-\bar{X}}{\bar{X}}, \end{array}$$

(9) $$\begin{array}{c} \theta_{1}=\left(\frac{1}{r}-\frac{1}{N}\right),\\ \theta_{2}=\left(\frac{1}{n}-\frac{1}{N}\right),\\ \theta_{3}=\left(\frac{1}{r}-\frac{1}{n}\right). \end{array}$$

The error terms have the following expectations:

(10) $$\begin{array}{c} E\left(\eta_{0}\right)=E\left(\eta_{1}\right)=E\left(\eta_{2}\right)=0,\\ E\left(\eta_{0}^{2}\right)=\theta_{1}C_{Y}^{2},E\left(\eta_{1}^{2}\right)=\theta_{1}C_{X}^{2},E\left(\eta_{2}^{2}\right)=\theta_{2}C_{X}^{2},\\ E\left(\eta_{0}\eta_{1}\right)=\theta_{1}\rho C_{Y}C_{X},E\left(\eta_{1}\eta_{2}\right)=\theta_{2}C_{X}^{2},E\left(\eta_{0}\eta_{2}\right)=\theta_{2}\rho C_{Y}C_{X}. \end{array}$$

To obtain the expressions for Bias and MSE, in the first step, the transformations in Equation (8) are applied to Equations (5)–(7). In the second step, algebraic expansion of the resultant expressions are done, using the following Taylor's series: log(1+*x*)=*x* − (*x*^2^/2)+((*x*^2^/2)/3) − ⋯.

The estimators take the following forms after algebraic manipulation:

(11) $$\begin{array}{c} T_{1}=\bar{Y}\left(1+\eta_{0}\right)+\alpha \left[\eta_{2}-\frac{\eta_{2}^{2}}{2}+\frac{\eta_{2}^{3}}{3}-\cdots \right],\\ T_{2}=\bar{Y}\left(1+\eta_{0}\right)+\beta \left[\eta_{1}-\frac{\eta_{1}^{2}}{2}+\frac{\eta_{1}^{3}}{3}-\cdots \right],\\ T_{3}=\bar{Y}\left(1+\eta_{0}\right)+\gamma \left[\left(\eta_{2}-\eta_{1}+\eta_{1}^{2}-\eta_{1}\eta_{2}\right)-\frac{{\left(\eta_{2}-\eta_{1}+\eta_{1}^{2}-\eta_{1}\eta_{2}\right)}^{2}}{2}+\cdots \right]. \end{array}$$

Hence,

(12) $$\begin{array}{c} T_{1}-\bar{Y}=\bar{Y}\eta_{0}+\alpha \left[\eta_{2}-\frac{\eta_{2}^{2}}{2}+\frac{\eta_{2}^{3}}{3}-\cdots \right],\\ T_{2}-\bar{Y}=\bar{Y}\eta_{0}+\beta \left[\eta_{1}-\frac{\eta_{1}^{2}}{2}+\frac{\eta_{1}^{3}}{3}-\cdots \right],\\ T_{3}-\bar{Y}=\bar{Y}\eta_{0}+\gamma \left[\left(\eta_{2}-\eta_{1}+\eta_{1}^{2}-\eta_{1}\eta_{2}\right)-\frac{{\left(\eta_{2}-\eta_{1}+\eta_{1}^{2}-\eta_{1}\eta_{2}\right)}^{2}}{2}+\cdots \right]. \end{array}$$

Expectations taken on the square of both sides yield the expressions for MSEs (*M*(.)). They are obtained up to the first order of approximations of the estimators *T*_*i*_, *i*=1,2,3, as follows:

(13) $$\begin{array}{c} M\left(T_{1}\right)=\theta_{1}S_{Y}^{2}+2\alpha \theta_{2}\rho S_{Y}C_{X}+\alpha^{2}\theta_{2}C_{X}^{2},\\ M\left(T_{2}\right)=\theta_{1}S_{Y}^{2}+2\beta \theta_{1}\rho S_{Y}C_{X}+\beta^{2}\theta_{1}C_{X}^{2},\\ M\left(T_{3}\right)=\theta_{1}S_{Y}^{2}-2\gamma \theta_{3}\rho S_{Y}C_{X}+\gamma^{2}\theta_{3}C_{X}^{2}. \end{array}$$

As stated when introducing the imputation methods, the constants *α*, *β*, and *γ* are to be determined so that they minimize the respective MSEs of the estimators. Setting

(14) $$\begin{array}{c} \frac{\partial M\left(T_{1}\right)}{\partial \alpha }=0,\frac{\partial M\left(T_{2}\right)}{\partial \beta }=0,\frac{\partial M\left(T_{3}\right)}{\partial \gamma }=0, \end{array}$$

the respective optimal values of *α*, *β*, and *γ* are obtained as follows:

(15) $$\begin{array}{c} \alpha_{\text{opt}}=-\rho \frac{S_{Y}}{C_{X}},\beta_{\text{opt}}=-\rho \frac{S_{Y}}{C_{X}},\gamma_{\text{opt}}=\rho \frac{S_{Y}}{C_{X}}. \end{array}$$

Thus, the expressions for the minimum MSE (Min M(.)) of the proposed classes of logarithmic-type estimators under optimal conditions are as follows:

(16) $$\begin{array}{c} \text{Min }M\left(T_{1}\right)=S_{Y}^{2}\left(\theta_{1}-\theta_{2}\rho^{2}\right), \end{array}$$

(17) $$\begin{array}{c} \text{Min }M\left(T_{2}\right)=\theta_{1}S_{Y}^{2}\left(1-\rho^{2}\right), \end{array}$$

(18) $$\begin{array}{c} \text{Min }M\left(T_{3}\right)=S_{Y}^{2}\left(\theta_{1}-\theta_{3}\rho^{2}\right). \end{array}$$

The expressions for bias *B*(.), using the optimal values of *α*, *β*,  and *γ*, are found to be as follows:

(19) $$\begin{array}{c} B\left(T_{1}\right)=\frac{\theta_{2}}{2}\rho S_{Y}C_{X},\\ B\left(T_{2}\right)=\frac{\theta_{1}}{2}\rho S_{Y}C_{X},\\ B\left(T_{1}\right)=\frac{\theta_{3}}{2}\rho S_{Y}C_{X}. \end{array}$$

Remark on practicability: a primary problem in the use of the proposed classes of logarithmic-type estimators *T*_*i*_, *i*=1,2,3, is the choice of the constants *α*, *β*, and *γ*. The optimum value of *α*, *β*, and *γ* depends on the population parameter *ρ*(*S*_*Y*_/*C*_*X*_). These values are seen to be overall stable when surveys are conducted repeatedly (see [30]); however, sometimes, the values remain unknown. In situations like that, the following estimators of *α*, *β*, and *γ* are suggested:

(20) $$\begin{array}{c} \hat{\alpha }=-r\frac{s_{yr}}{c_{xr}},\hat{\beta }=-r\frac{s_{yr}}{c_{xr}},\hat{\gamma }=r\frac{s_{yr}}{c_{xr}}, \end{array}$$

where *r* is the correlation coefficient between *X* and *Y*, *s*_*yr*_ is the sample mean square of *Y*, and *c*_*xr*_ is the sample coefficient of variation of *X*, based on the responding part of the sample of size *r*.

### 4.3. Implementation in R

In today's technologically advanced world, most computations are done in some suitable software environment. The *R* [31] code snippet given in the following can be used to carry out the proposed imputations on a dataset of interest and calculate the values of the corresponding point estimators:

  #Import data of respondents from file
  dfresp < - read.table(file.choose())
  #Import data of non-respondents from file
  dfnonresp < - read.table(file.choose())
  xrbar = mean(dfresp[,1])
  yrbar < -mean(dfresp[,2])
  xbar < - XXX #Specify known value of Xbar here
  rhosamp = corr(dfresp[,1],dfresp[,2])
  sxr = var(dfresp[,1])
  syr = var(dfresp[,2])
  cyr = syr/yrbar
  cxr = sxr/xrbar
  xbarnonresp = mean(dfnonresp[,1])
 
*r* = nrow(dfresp) #no. of respondents
  nonresp = nrow(dfnonresp) #no. of non-respondents
 
*n* = *r* + nonresp #sample size
  xnbar=(*r*^*∗*^xrbar + nonresp^*∗*^xbarnonresp)/*n*
  const = rhosamp^*∗*^syr/cxr
  alpha = -const
  beta = -const
  gamma = const
  #imputation
 
*t*1<−c()
 
*t*2<−c()
 
*t*3<−c()
  for(i in 1:(*n* − *r*))
  {
 
*t*1[*i*] = yrbar + alpha^*∗*^*n*^*∗*^*x*[*i*]^*∗*^log(xnbar/Xbar)/((*n* − *r*)^*∗*^xbarnonresp)
 
*t*2[*i*] = yrbar + beta^*∗*^*n*^*∗*^x[*i*]^*∗*^log(xrbar/Xbar)/((*n* − *r*)^*∗*^xbarnonresp)
 
*t*3[*i*] = yrbar + gamma^*∗*^*n*^*∗*^*x*[*i*]^*∗*^log(xnbar/xrbar)/((*n* − *r*)^*∗*^xbarnonresp)
  }
  #point estimation
  est1 = yrbar + alpha^*∗*^log(xnbar/Xbar)
  est2 = yrbar + beta^*∗*^log(xrbar/Xbar)
  est3 = yrbar + gamma^*∗*^log(xnbar/xrbar)

## 5. Empirical Study

Before an estimator can be used in practical scenarios, its performance must be examined, in terms of its properties. To this end, the biases of the estimators are calculated and the MSEs under optimal conditions are compared with those of the contemporary estimators given in Table 2 within the framework of percentage relative efficiencies (PREs).

The PREs of the classes of logarithmic-type estimators w.r.t. the contemporary estimators, under optimal conditions, are defined as follows:

(21) $$\begin{array}{c} PRE_{i1}=\frac{V\left({\bar{y}}_{m}\right)}{\text{Min}M\left(T_{i}\right)}\times 100,\;i=1,2,3,\\ PRE_{i2}=\frac{M\left({\bar{y}}_{RAT}\right)}{\text{Min}M\left(T_{i}\right)}\times 100,\;i=1,2,3,\\ PRE_{i3}=\frac{M\left(T_{TSS}\right)}{\text{Min}M\left(T_{i}\right)}\times 100,\;i=1,2,3,\\ PRE_{i4}=\frac{M\left(T_{KC_{1}}\right)}{\text{Min}M\left(T_{i}\right)}\times 100,\;i=1,2,3,\\ PRE_{i5}=\frac{M\left(T_{KC_{2}}\right)}{\text{Min}M\left(T_{i}\right)}\times 100,\;i=1,2,3,\\ PRE_{i6}=\frac{M\left(T_{KC_{3}}\right)}{\text{Min}M\left(T_{i}\right)}\times 100,\;i=1,2,3. \end{array}$$

Here, the expressions for the Min. MSEs of the proposed classes of logarithmic-type estimators *T*_*i*_,  *i*=1,2,3, are given in Equations (16)–(18), while those of the contemporary estimators are given in Table 3.

Using *R* [31], an extensive simulation study has been carried out on sufficiently large fictitious populations to compute the biases and the PREs defined above. Data is generated from three different probability distributions, namely, normal (a continuous distribution), Poisson (a discrete distribution), and Gamma (a continuous distribution) distributions. A few important properties of the distributions have been tabulated in Table 4. Such distributions have been selected because they are frequently seen to occur in real-life situations.

Normal distribution has uses in modeling of heights of individuals, test scores of students, blood pressure, daily returns of any particular stock, weights of items produced by a manufacturing process, etc. Poisson distribution can be used to model the probability that a given number of events occur in a specific time interval, for example, the number of insurance claims filed per month, the number of network failures occurring per week, and the number of bulbs manufactured per minute. It also finds use in medical statistics, such as for estimating the number of births that may be expected on a particular night, the number of patients with an infectious disease arriving at a clinic within a given hour, and the number of mutations on a given strand of DNA per time unit. Gamma distribution can be used for modeling wait time, reliability, service time in queuing theory, etc. For example, it can be used to model the amount of rainfall that accumulates in a given reservoir, the flow of items through manufacturing as well as distribution processes, the size of loan defaults, etc. Thus, these three distributions are chosen based on their importance in practical scenarios.

The steps of the simulation are as follows:

1. The sizes of the population, the sample, and the responding part of the sample are defined. For the purpose of the study, sufficiently large values of *N*=100000, *n*=40000, and *r*=35000 have been chosen.
2. The parameters of the population are defined. Data is generated from normal distribution with parameters *N*(10,1) for *X* and *N*(12,1) for *X*, from Gamma distribution with parameters with means 3,5 and variances 1,1 for *X* and *Y*, respectively, and from Poisson distribution with means 10,12 for *X* and *Y*, respectively.
3. Simulation is conducted for various values of *ρ*. For the purpose of the study, *ρ* in the range (0.1, 0.9), i.e., positively correlated variable *X*, is considered.

The results of the simulation study related to the PREs have been presented in Tables 5–13, while the biases have been presented in Tables 14–16.

## 6. Application to Real Data

Secondary data has been used for the purpose of demonstrating the utilization of the proposed estimator under the SRSWOR sampling scheme. The dataset “Chemical Composition of Ceramic Samples Data Set” has been obtained from UCI Machine Learning Repository [32] and used to illustrate the use of the proposed estimator in real-world scenarios for estimating population mean. The dataset consists of 88 instances of 19 attributes and is concerned with the classification of ceramic samples depending on their chemical composition from energy-dispersive X-ray fluorescence. We use the subset of the dataset where attribute “Part” takes the value “Body,” so that *N*=44. Here,

 
*X*: percentage of MgO (wt%)
 
*Y*: percentage of CaO (wt%)

**Figure 1:**
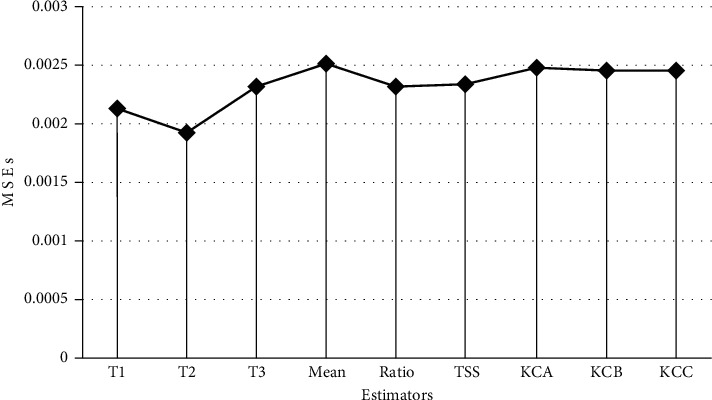
MSEs of the proposed estimators and the contemporary estimators for the real dataset.

It is seen that *ρ*=0.4880444. Taking *n*=18 and *r*=14, the PREs are found to be as given in Table 17. The MSEs of the proposed estimators and the contemporary estimators have been plotted in Figure 1.

## 7. Conclusions

The empirical study enables us to study the behavior of the proposed estimator under various scenarios involving various values of parameters. The chief conclusions that follow are given next:

1. Tables 5–7 show that the proposed classes of logarithmic-type estimators *T*_*i*_, *i*=1,2,3, are more efficient than the contemporary estimators when data is generated from normal distribution.
2. The PRE of the proposed classes of estimators w.r.t. the contemporary estimators is seen to increase with the increase in the value of *ρ*, i.e., the correlation coefficient between the study and the auxiliary variables, as evident from Tables 5–7.
3. From Tables 8–10, it is observed that the proposed classes of logarithmic-type estimators *T*_*i*_, *i*=1,2,3, dominate over the contemporary estimators when data is generated from Gamma distribution.
4. The proposed estimators *T*_*i*_, *i*=1,2,3, perform better than the contemporary estimators in terms of PREs when data is generated from Poisson distribution, as seen from Tables 11–13.
5. Tables 14–16 show that the biases of the proposed estimators are negligible, being of orders 10^−6^ and 10^−7^, when data is generated from normal, Gamma, and Poisson distributions, respectively.
6. Table 17 shows that for the real data used in this manuscript, the classes of logarithmic-type estimators proposed in the manuscript dominate over the contemporary estimators for situations when the variables *X* and *Y* have a moderate positive value of the correlation coefficient. Furthermore, from Figure 1, it is graphically seen that the MSEs of the proposed estimators *T*_*i*_, *i*=1,2,3, are less than that of the contemporary estimators.

Hence, the proposed estimator is seen to be consistent, exists for all real positive values of parameters, has negligible bias, and is more efficient than 6 other contemporary estimators. Hence, the proposed estimator may be recommended for use in field work.

## Figures and Tables

**Table 1:** Sample structure and notations.

Structure	Size
Population	*N*
Sample	*n*
Respondents	*r*
Nonrespondents	*n*-*r*
Characteristic	Notation
The population mean of Y	$\bar{Y}$
The population mean of *X*	$\bar{X}$
The sample mean of *Y* based on the responding part of the sample	${\bar{y}}_{r}$
The sample mean of *X* based on the responding part of the sample	${\bar{x}}_{r}$
The sample means of *X*, respectively, based on the entire sample	${\bar{x}}_{n}$
The correlation coefficient between *X* and *Y*	*ρ*
The population mean square of *X*	*S*_*X*_^2^
The population mean square of *Y*	*S*_*Y*_^2^
The coefficient of variation of *X*	*C*_*X*_
The coefficient of variation *Y*	*C*_*Y*_

**Table 2:** Structures of some well-known estimators.

Estimator	Notation used	Structure
Mean estimator	${\bar{y}}_{m}$	${\bar{y}}_{r}$
Ratio estimator	${\bar{y}}_{RAT}$	${\bar{y}}_{r}\frac{{\bar{x}}_{n}}{{\bar{x}}_{r}}$
Kadilar and Cingi [29] estimator A	*T*_*KC*_*A*__	$\left(\left({\bar{y}}_{r}+b\left(\bar{X}-{\bar{x}}_{n}\right)\right)/{\bar{x}}_{n}\right)\bar{X}$
Kadilar and Cingi [29] estimator B	*T*_*KC*_*B*__	$\left(\left({\bar{y}}_{r}+b\left(\bar{X}-{\bar{x}}_{r}\right)\right)/{\bar{x}}_{r}\right)\bar{X}$
Kadilar and Cingi [29] estimator C	*T*_*KC*_*C*__	$\left(\left({\bar{y}}_{r}+b\left({\bar{x}}_{n}-{\bar{x}}_{n}\right)\right)/{\bar{x}}_{r}\right)\bar{X}$
Toutenberg and Srivastava [28] estimator	*T*_*TSS*_	${\bar{y}}_{r}+\left(r/n\right)\left({\bar{y}}_{r}/{\bar{x}}_{n}\right)\left({\bar{x}}_{n}-{\bar{x}}_{r}\right)$

**Table 3:** MSEs of some well-known estimators.

Estimator	Variance (V) or Mean Square Error (MSE)
${\bar{y}}_{m}$	$V\left({\bar{y}}_{m}\right)=\theta_{1}S_{Y}^{2}$
${\bar{y}}_{RAT}$	$MSE\left({\bar{y}}_{RAT}\right)=\theta_{2}S_{Y}^{2}+\theta_{3}\left(S_{Y}^{2}+R_{1}^{2}S_{X}^{2}-2R_{1}\rho S_{Y}S_{X}\right)$
*T*_*KC*_*A*__	*MSE*(*T*_*KC*_*A*__)=((1/*r*) − (1/*N*))*S*_*Y*_^2^+((1/*n*) − (1/*N*))*S*_*X*_^2^(*R*_1_^2^ − *B*^2^)
*T*_*KC*_*B*__	*MSE*(*T*_*KC*_*B*__)=((1/*r*) − (1/*N*))(*S*_*Y*_^2^ − *BS*_*YX*_+*R*^2^*S*_*X*_^2^)
*T*_*KC*_*C*__	*MSE*(*T*_*KC*_*C*__)=((1/*r*) − (1/*N*))*S*_*Y*_^2^+((1/*r*) − (1/*N*))((*R*+*B*)^2^*S*_*X*_^2^ − 2(*R*+*B*)*S*_*XY*_)
*T*_*TSS*_	$MSE\left(T_{TSS}\right)=\left(\left(1/r\right)-\left(1/N\right)\right)S_{Y}^{2}+{\bar{Y}}^{2}\left(\left(1/r\right)-\left(1/n\right)\right)\left(r/n\right)\left(\left(r/n\right)C_{X}^{2}-2\rho C_{Y}C_{X}\right)$
	Where $R_{1}=\left(\bar{Y}/\bar{X}\right)$, *B*=(*S*_*XY*_/*S*_*X*_^2^)

**Table 4:** Some properties of normal, Poisson, and Gamma distributions.

Distribution	Normal
Parameters	*μ*, *σ*^2^
Pdf	$f\left(x\right)=\left(1/\sigma \sqrt{2\pi }\right)\exp\left[-\left(\left({\left(x-\mu \right)}^{2}\right)/2\sigma^{2}\right)\right],-\infty <x<\infty$
Mean *E*(*X*)	*μ*
Variance *V*(*X*)	*σ* ^2^

Distribution	Poisson
Parameter	*λ* > 0
Pmf	*f*(*x*)=(*λ*^*x*^*e*^−*λ*^/*x*!)
Mean *E*(*X*)	*λ*
Variance *V*(*X*)	*λ*

Distribution	Gamma
Parameters	*α*, *λ*
Pdf	$f\left(x\right)=\left\{\begin{array}{c} \left(\lambda^{\alpha }x^{\alpha -1}e^{-\lambda x}/\Gamma \left(x\right)\right)\text{, if }x>0\\ 0\text{, otherwise} \end{array}\right.$
Mean *E*(*X*)	(*α*/*λ*)
Variance *V*(*X*)	(*α*/*λ*^2^)

**Table 5:** Values of *PRE*_1*i*_, *i*=1,2,3,4,5,6, when data is generated from normal distribution.

*ρ*	*PRE*_11_	*PRE*_12_	*PRE*_13_	*PRE*_14_	*PRE*_15_	*PRE*_16_
0.1	100.8207	124.1350	118.1591	217.5655	245.3619	245.3619
0.2	103.4355	122.9348	117.2939	223.0118	251.4823	251.4823
0.3	108.0039	122.8726	117.7341	225.9495	254.0318	254.0318
0.4	115.1905	125.8011	120.9539	235.2020	263.7762	263.7762
0.5	124.6105	130.5359	126.0475	243.9122	272.3174	272.3174
0.6	141.3373	141.3371	137.0312	265.3476	294.8739	294.8739
0.7	164.8015	157.4136	153.3335	292.1363	322.4542	322.4542
0.8	208.1384	188.7934	184.8866	342.8744	374.9544	374.9544
0.9	291.9545	251.1717	247.4521	438.5919	473.5056	473.5056

**Table 6:** Values of *PRE*_2*i*_, *i*=1,2,3,4,5,6, when data is generated from normal distribution.

*ρ*	*PRE*_21_	*PRE*_22_	*PRE*_23_	*PRE*_24_	*PRE*_25_	*PRE*_26_
0.1	101.0181	124.3780	118.3904	217.9915	245.8423	245.8423
0.2	104.2886	123.9487	118.2613	224.8510	253.5564	253.5564
0.3	110.1021	125.2597	120.0213	230.3391	258.9669	258.9669
0.4	119.5130	130.5218	125.4927	244.0280	273.6744	273.6744
0.5	132.3667	138.6610	133.8932	259.0942	289.2674	289.2674
0.6	156.7666	156.7664	151.9904	294.3147	327.0643	327.0643
0.7	194.8675	186.1317	181.3073	345.4330	381.2819	381.2819
0.8	280.3108	254.2577	248.9963	461.7666	504.9703	504.9703
0.9	537.7034	462.5924	455.7418	807.7712	872.0730	872.0730

**Table 7:** Values of *PRE*_3*i*_, *i*=1,2,3,4,5,6, when data is generated from normal distribution.

*ρ*	*PRE*_31_	*PRE*_32_	*PRE*_33_	*PRE*_34_	*PRE*_35_	*PRE*_36_
0.1	100.1942	123.3636	117.4248	216.2135	243.8372	243.8372
0.2	100.7971	119.7990	114.3020	217.3233	245.0676	245.0676
0.3	101.7962	115.8103	110.9671	212.9626	239.4308	239.4308
0.4	103.2416	112.7516	108.4072	210.8042	236.4143	236.4143
0.5	104.9344	109.9242	106.1446	205.3983	229.3183	229.3183
0.6	107.4849	107.4847	104.2101	201.7928	224.2471	224.2471
0.7	110.3292	105.3832	102.6517	195.5756	215.8724	215.8724
0.8	114.1165	103.5101	101.3682	187.9884	205.5770	205.5770
0.9	118.5597	101.9983	100.4877	178.1077	192.2858	192.2858

**Table 8:** Values of *PRE*_1*i*_, *i*=1,2,3,4,5,6, when data is generated from Gamma distribution.

*ρ*	*PRE*_11_	*PRE*_12_	*PRE*_13_	*PRE*_14_	*PRE*_15_	*PRE*_16_
0.1	100.5437	116.8810	112.7042	181.9684	201.3552	201.3552
0.2	102.4607	115.6733	111.8264	184.2976	203.7826	203.7826
0.3	106.1375	115.6772	112.2446	186.0215	205.0414	205.0414
0.4	111.7249	117.9257	114.7657	191.5801	210.5932	210.5932
0.5	119.8807	121.9171	119.1661	195.8653	213.9569	213.9569
0.6	133.5989	130.6786	128.2187	208.4785	226.3069	226.3069
0.7	154.4129	144.9483	142.8615	225.5645	242.5054	242.5054
0.8	193.7043	173.3931	171.8121	258.2036	273.5605	273.5605
0.9	271.5579	231.4410	230.7134	320.5030	332.1566	332.1566

**Table 9:** Values of *PRE*_2*i*_, *i*=1,2,3,4,5,6, when data is generated from Gamma distribution.

*ρ*	*PRE*_21_	*PRE*_22_	*PRE*_23_	*PRE*_24_	*PRE*_25_	*PRE*_26_
0.1	100.6741	117.0325	112.8503	182.2043	201.6162	201.6162
0.2	103.0645	116.3549	112.4854	185.3837	204.9835	204.9835
0.3	107.7115	117.3927	113.9092	188.7801	208.0822	208.0822
0.4	114.9334	121.3123	118.0615	197.0819	216.6411	216.6411
0.5	125.8373	127.9748	125.0871	205.5973	224.5878	224.5878
0.6	145.2157	142.0415	139.3677	226.6064	245.9851	245.9851
0.7	177.3953	166.5220	164.1246	259.1369	278.5992	278.5992
0.8	249.3315	223.1874	221.1524	332.3534	352.1205	352.1205
0.9	459.0779	391.2590	390.0289	541.8213	561.5222	561.5222

**Table 10:** Values of *PRE*_3*i*_, *i*=1,2,3,4,5,6, when data is generated from Gamma distribution.

*ρ*	*PRE*_31_	*PRE*_32_	*PRE*_33_	*PRE*_34_	*PRE*_35_	*PRE*_36_
0.1	100.1289	116.3988	112.2393	181.2177	200.5245	200.5245
0.2	100.5751	113.5445	109.7685	180.9060	200.0324	200.0324
0.3	101.3960	110.5096	107.2303	177.7114	195.8817	195.8817
0.4	102.5627	108.2550	105.3541	175.8693	193.3232	193.3232
0.5	104.1108	105.8793	103.4902	170.0999	185.8116	185.8116
0.6	106.3693	104.0442	102.0856	165.9872	180.1819	180.1819
0.7	109.1586	102.4678	100.9925	159.4575	171.4334	171.4334
0.8	113.0171	101.1665	100.2441	150.6494	159.6094	159.6094
0.9	117.7049	100.3165	100.0011	138.9198	143.9710	143.9710

**Table 11:** Values of *PRE*_1*i*_, *i*=1,2,3,4,5,6, when data is generated from Poisson distribution.

*ρ*	*PRE*_11_	*PRE*_12_	*PRE*_13_	*PRE*_14_	*PRE*_15_	*PRE*_16_
0.1	100.8736	119.9006	114.9581	198.4622	221.6976	221.6976
0.2	103.3705	118.4010	113.9229	199.8112	222.7732	222.7732
0.3	107.7667	119.0086	114.8930	204.0789	227.0104	227.0104
0.4	113.8133	121.5329	117.6879	210.5924	233.6351	233.6351
0.5	125.0182	127.4591	124.0369	219.6983	242.2412	242.2412
0.6	139.5973	136.9348	133.7494	235.0984	257.8367	257.8367
0.7	164.6748	154.1583	151.3579	258.0149	280.2387	280.2387
0.8	204.1033	182.8606	180.3672	297.7136	320.0018	320.0018
0.9	281.7346	240.6873	238.6971	373.4524	395.2899	395.2899

**Table 12:** Values of *PRE*_2*i*_, *i*=1,2,3,4,5,6, when data is generated from Poisson distribution.

*ρ*	*PRE*_21_	*PRE*_22_	*PRE*_23_	*PRE*_24_	*PRE*_25_	*PRE*_26_
0.1	101.0839	120.1505	115.1977	198.8759	222.1597	222.1597
0.2	104.2068	119.3588	114.8446	201.4276	224.5755	224.5755
0.3	109.7971	121.2508	117.0576	207.9239	231.2874	231.2874
0.4	117.6837	125.6658	121.6901	217.7541	241.5803	241.5803
0.5	132.9369	135.5324	131.8934	233.6141	257.5848	257.5848
0.6	154.1285	151.1888	147.6718	259.5705	284.6758	284.6758
0.7	194.6481	182.2176	178.9074	304.9776	331.2465	331.2465
0.8	271.3653	243.1221	239.8069	395.8247	425.4579	425.4579
0.9	496.6249	424.2693	420.7610	658.2995	696.7934	696.7934

**Table 13:** Values of *PRE*_3*i*_, *i*=1,2,3,4,5,6, when data is generated from Poisson distribution.

*ρ*	*PRE*_31_	*PRE*_32_	*PRE*_33_	*PRE*_34_	*PRE*_35_	*PRE*_36_
0.1	100.1838	119.3312	114.3910	197.2077	220.3087	220.3087
0.2	100.7721	115.9988	111.4924	197.4902	220.5183	220.5183
0.3	101.7553	112.4365	108.5271	193.2357	215.0168	215.0168
0.4	103.1652	109.8054	106.3381	191.1441	212.0915	212.0915
0.5	104.8369	107.3226	104.3801	185.5838	204.8093	204.8093
0.6	107.3491	105.1686	102.7238	180.8278	198.3227	198.3227
0.7	110.1095	103.5049	101.5393	174.8319	190.2419	190.2419
0.8	113.8590	102.0032	100.5976	166.5320	179.0732	179.0732
0.9	118.2216	100.9056	100.0894	156.1465	165.1762	165.1762

**Table 14:** Values of *B*(*T*_*i*_), *i*=1,2,3, for data simulated from normal distribution.

*ρ*	*B*(*T*_1_)	*B*(*T*_2_)	*B*(*T*_3_)
0.1	0.000000000	0.000000000	0.000000000
0.2	0.000000000	0.000000000	0.000000000
0.3	0.000000000	0.000000000	0.000000000
0.4	0.000000000	0.000000000	0.000000000
0.5	0.000000000	0.000000000	0.000000000
0.6	0.000000000	0.000001000	0.000000000
0.7	0.000001000	0.000001000	0.000000000
0.8	0.000001000	0.000001000	0.000000000
0.9	0.000001000	0.000001000	0.000000000

**Table 15:** Values of *B*(*T*_*i*_), *i*=1,2,3, for data simulated from Gamma distribution.

*ρ*	*B*(*T*_1_)	*B*(*T*_2_)	*B*(*T*_3_)
0.1	0.000003240	0.000004010	0.000000770
0.2	0.000006470	0.000008010	0.000001540
0.3	0.000009960	0.000012330	0.000002370
0.4	0.000013400	0.000016590	0.000003190
0.5	0.000016650	0.000020610	0.000003960
0.6	0.000021350	0.000026430	0.000005080
0.7	0.000024060	0.000029790	0.000005730
0.8	0.000028700	0.000035540	0.000006830
0.9	0.000033500	0.000041480	0.000007980

**Table 16:** Values of *B*(*T*_*i*_), *i*=1,2,3, for data simulated from Poisson distribution.

*ρ*	*B*(*T*_1_)	*B*(*T*_2_)	*B*(*T*_3_)
0.1	0.000000828	0.000001026	0.000000197
0.2	0.000001651	0.000002044	0.000000393
0.3	0.000002523	0.000003123	0.000000601
0.4	0.000003280	0.000004061	0.000000781
0.5	0.000004092	0.000005067	0.000000974
0.6	0.000004944	0.000006121	0.000001177
0.7	0.000005703	0.000007061	0.000001358
0.8	0.000006627	0.000008205	0.000001578
0.9	0.000007342	0.000009090	0.000001748

**Table 17:** Values of *PRE*_*ij*_, *i*=1,2,3 and *j*=1,2,3,4,5,6, for real data.

*i*	Estimator	*PRE*_*i*1_	*PRE*_*i*2_	*PRE*_*i*3_	*PRE*_*i*4_	*PRE*_*i*5_	*PRE*_*i*6_
1	*T*_1_	119.1265	109.9138	110.5542	116.8349	115.7269	115.7269
2	*T*_2_	131.2659	121.1144	121.8200	128.7408	127.5199	127.5199
3	*T*_3_	108.4165	100.0322	100.6149	106.3310	105.3226	105.3226

## Data Availability

The data used in the study are generated theoretically by the equations given in this paper.
